# Can we help?

**DOI:** 10.4103/0972-124X.65441

**Published:** 2010

**Authors:** Rajvir Malik

**Affiliations:** *Department of Periodontics and Implantology, DAV (C) Dental College and Hospital, Yamuna Nagar, Haryana - 135 001, India. E-mail: rajvirmalik@yahoo.com*

It was really good to read the editorial entitled “The first word” by Dr. Arunachalam.[1] The axiom “the more things change, the more they stay the same,” has been engrossed very wonderfully in the pretext of Periodontology and its practice today.

Recently, I was going through the editorial titled “Dental education in India,” by Dr. Sivapathasundharam[2] in which he says that it is the moral responsibility of the Central Government and the DCI to ensure the future of thousands of BDS students graduating from various recognized institutions every year. In another guest editorial, Dr. Anil Kohli[3] asserted that this is undoubtedly an exciting time in dentistry and the biomedical community at large. In 20 to 25 years, dentistry, as we know it today, will be remarkably different, as it is now different from the way it was 25 years ago.

No doubt both my senior colleagues are quite right in their own annotations. But one question which usually arises and baffles all is why there has been no specific answer to the query - where do we stand in regard to the curriculum of under graduate students? During our times and even before that, Glickman’s Clinical Periodontology was the standard book followed. But with time and increase in the in-depth knowledge of the subject, the recent editions of Carranza’s Clinical Periodontology seem to be beyond the scope of an under graduate student. Also available are a whole lot of Periodontology text books by Indian as well as foreign authors. This makes the task of a student more daunting in choosing the appropriate textbook. Even more is difficult for a teacher to suggest a particular textbook to his/her students. Post Graduate students have a whole three years time to go through various books and journals to understand and formulate their own concepts regarding the subject. It is the quandary of an under graduate student, who has to deal with six- seven subjects of Dentistry in just one year. Now the question arises - who will take the initiative to get to the bottom of this dilemma?

In this context I really appreciate the debate started by Dr. Sivapathasundharam[4] and Dr. Baskar[5] to do away with tooth carving exercise in the subject of Dental Anatomy.

My real poser to the highly dignified and proficient Periodontal as well as other faculties in various specialties is - can we do something for the future of these budding dental professionals to make their sailing a bit more smooth and uncomplicated?
